# Rural patients' satisfaction with humanistic nursing care in Chinese Public Tertiary Hospitals: a national cross-sectional study

**DOI:** 10.3389/fpubh.2024.1455305

**Published:** 2024-12-19

**Authors:** Chuang Li, Ning Wang, Haixin Zhang, Yongguang Yan, Huiling Chen, Ruxin Jiang, Yulan Chang, Pingfan Zhao, Qiaomei Cheng, Bing Song, Shujie Guo

**Affiliations:** ^1^Henan Provincial People's Hospital, Zhengzhou University People's Hospital, Zhengzhou, Henan, China; ^2^Outpatient Department, Henan Provincial People's Hospital, Zhengzhou, Henan, China; ^3^Department of Orthopedic, Qilu Hospital of Shandong University Dezhou Hospital, Dezhou, Shandong, China; ^4^Department of Nursing, Henan Provincial People's Hospital, Zhengzhou, Henan, China; ^5^Research and Foreign Affairs Department, Henan Provincial People's Hospital, Zhengzhou, Henan, China; ^6^Heart Center of Henan Provincial People's Hospital, Central China Fuwai Hospital, Central China Fuwai Hospital of Zhengzhou University, Zhengzhou, Henan, China; ^7^Department of Anesthesiology and Perioperative Medicine, Henan Provincal People's Hospital, Zhengzhou, Henan, China; ^8^Department of Nursing, Henan Vocational College of Nursing, Anyang, Henan, China; ^9^South Henan Branch of Henan Provincial People's Hospital, Xinyang, Henan, China

**Keywords:** rural patients, humanistic nursing care, Chinese Public Tertiary Hospitals, patient satisfaction, cross-sectional study

## Abstract

**Background:**

The provision of high-quality healthcare services and patient satisfaction are fundamental objectives in modern healthcare. Humanistic nursing care, which emphasizes empathy, respect for individuality, and cultural sensitivity, aims to build trust and improve the overall experience for patients. This approach is especially relevant for rural patients in China, who often face additional challenges in accessing care in large tertiary hospitals.

**Methods:**

A multistage, stratified sampling method was employed to collect data from 8,263 patients aged 18 years or older in large public tertiary hospitals. Humanistic care satisfaction scores were measured using the Nurse Caring Instrument (NCI) questionnaire, a validated tool for assessing patient satisfaction with nursing care.

**Results:**

Satisfaction with nursing humanistic care among rural Chinese patients attending large tertiary public hospitals was low with the overall mean satisfaction score 81.62 ± 16.85. Significant differences in satisfaction were found based on age, marital status, number of children, educational attainment, occupation, monthly household income, department visited, type of medical insurance, and first-time visitor. A multivariate analysis revealed positive correlations with satisfaction for factors such as having children, higher education, higher family monthly income, and first-time visitor, and negative correlations for factors such as older age, being widowed, department visited, and region.

**Conclusion:**

Older adults, widowed individuals, and first-time patients expressed lower levels of satisfaction, highlighting the need for tailored interventions. The findings provide insights into the impact of humanistic nursing care for rural patients and emphasize the importance of culturally sensitive approaches to improve patient satisfaction in rural China. This study has several limitations. The cross-sectional design restricts the ability to establish causal relationships, and there is a potential for selection bias, as participants who completed the survey may have higher educational and economic levels, possibly leading to an overestimation of satisfaction. Lastly, as this study focused on rural patients in large public tertiary hospitals in China, the findings may not be generalizable to other settings or patient groups. Future studies should address these limitations for broader applicability and insight.

## 1 Introduction

The provision of high-quality healthcare services and patient satisfaction are the fundamental objectives of modern healthcare. High-quality healthcare services encompass a multifaceted spectrum that not only includes essential biomedical interventions, such as pharmacotherapy, surgical procedures, and standard nursing care, but also the broader dimensions of humanistic nursing care. The concept of humanistic care was initially proposed in the 1970s by Madeleine Leininger, an American nursing scholar, and subsequently developed and expanded by Jean Watson, who established the theoretical underpinnings of nursing humanism (1–3). The essence of humanistic nursing care lies in meeting patients' needs and enabling them to feel both physical and psychological support through nursing competencies, attitudes, and behaviors. Its primary goal is to help patients achieve physiological, psychological, and sociocultural wellbeing. Humanistic care is not only a professional attitude and emotional labor but also a dynamic process that integrates nursing procedures with nurse-patient communication. As one of the fundamental needs of patients, humanistic care is not only a critical component of patient satisfaction but also a key indicator and intrinsic element of high-quality nursing services. With the continuous progress in social development, humanistic nursing care is practiced by more and more healthcare facilities around the world. Guided by the International Circulation Care Association, 42 Humanistic Care Guidelines for nurses were introduced in 2003. In China, expert Liu et al. incorporated Watson's 10 caring elements into research on humanistic nursing practice for care management. Using theoretical guidance, pilot caring wards, thematic research, international collaboration, and industry exchanges, they established an inpatient humanistic care model and improved evaluation indicators, providing strategic direction for nursing managers (4, 5). Humanistic nursing care emphasizes the establishment of profound empathetic connections between healthcare providers and patients with a focus on compassion, empathy, and culturally sensitive interactions (6, 7). Humanistic nursing is vital for patients; it is an important aspect of patient satisfaction and an essential component of quality nursing care (8, 9). The absence of humanistic nursing has a direct impact on patient recovery, reduces the quality of medical services and patient satisfaction, and can lead to nurse–patient conflict (10–12).

Guided by the International Circulation Care Association, 42 Humanistic Care Guidelines for nurses were introduced in 2003 (16). That year, Watson developed the ANCM model from a patient-centered approach to strengthen nurses' caring abilities through programs covering care assessment, planning, implementation, and continuous care (17). In China, expert Liu et al. (18–22) incorporated Watson's 10 caring elements into research on humanistic nursing practice for care management. Using theoretical guidance, pilot caring wards, thematic research, international collaboration, and industry exchanges, they established an inpatient humanistic care model and improved evaluation indicators, providing strategic direction for nursing managers.

While China has experienced rapid urbanization in recent years, a significant portion of its population remains predominantly rural, with the majority residing in rural areas. Despite this demographic landscape, the distribution of healthcare infrastructure and resources in China heavily favors urban centers, leaving rural areas inadequacy of healthcare resources. According to official statistics released by the National Health and Family Planning Commission of China in 2017, the total urban healthcare expenditure in China increased from 262.42 billion RMB in 2000 to 2,657.56 billion RMB in 2014, representing a more than tenfold increase. During the same period, the proportion of urban healthcare expenditure in the total national healthcare expenditure rose from 57% to 75%. In contrast, rural healthcare expenditure grew from 196.24 billion RMB in 2000 to 873.68 billion RMB in 2014, an approximately fourfold increase. However, the proportion of rural healthcare expenditure in the total national healthcare expenditure declined from 43% to 25% over the same period.

Consequently, most rural patients often choose to go to large tertiary hospitals in regional centers when they face more serious illnesses (13, 14). At present, rural patients in China constitute a significant proportion of the population seeking healthcare services in large tertiary hospitals, these patients often undertake arduous journeys to access specialized care, facing a myriad of challenges, including arduous journeys to access healthcare facilities, economic disparities, communication difficulties and cultural differences, and limited healthcare resources. This psychological dilemma of the helplessness is especially pronounced when traveling to a large medical institution in a regional city (15, 16). In this context, the provision of humanistic nursing care has become essential to enhancing rural patients' overall healthcare experience and satisfaction. It not only addresses patients' physical needs but also their emotional and psychological requirements, contributing to the provision of more comprehensive healthcare services.

This study assesses rural patients' satisfaction with humanistic nursing care in large public tertiary hospitals in China, and seeks to identify areas for improvement to ensure that rural patients receive healthcare that not only addresses their clinical needs but also their emotional and psychological requirements.

## 2 Methods

### 2.1 Study design

**Figure 1:**
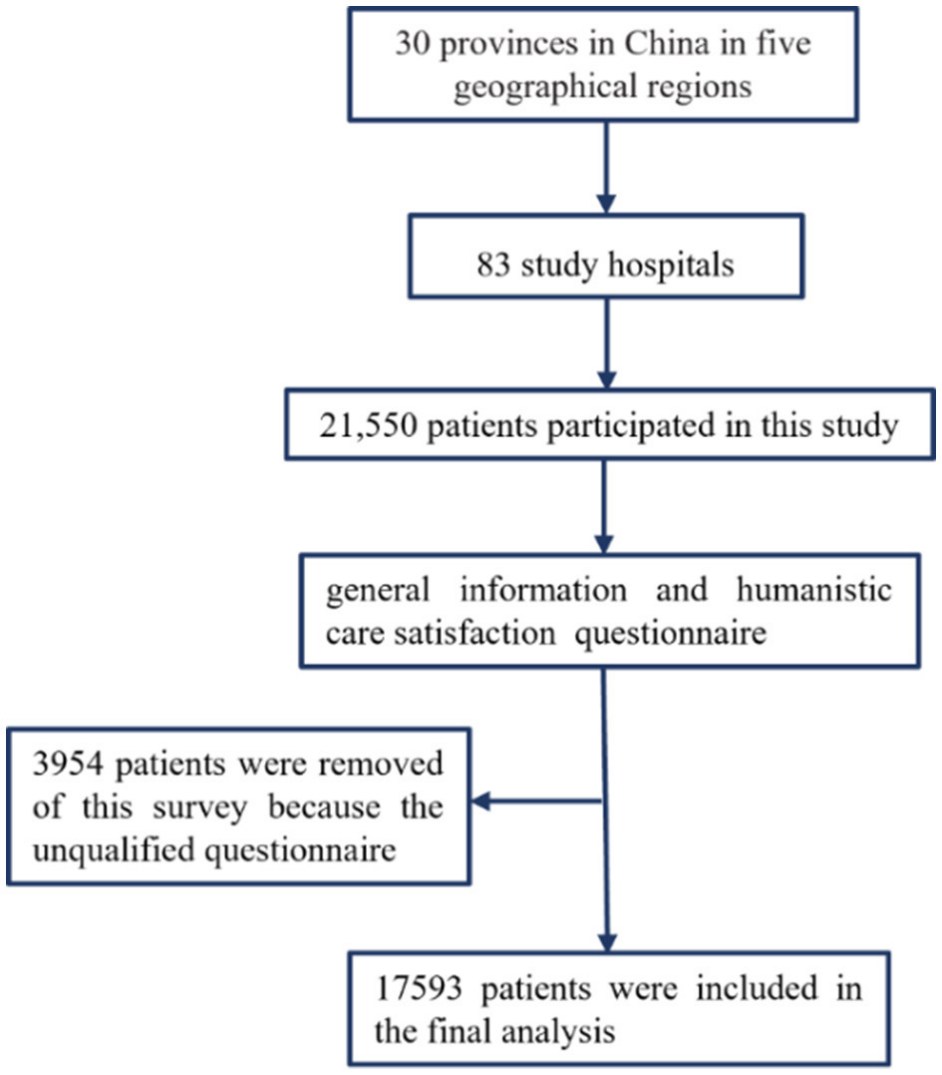
Hospital sites and respondent selection process.

The study protocol was approved by the Ethics Committee of Tongji Medical College, Huazhong University of Science and Technolog (Approval No.: 2022S161). This national cross-sectional study used a multistage, stratified sampling method to obtain a representative sample of people aged 18 years or older to assess patient-reported satisfaction with humanistic nursing care among rural patients in large public tertiary hospitals in China from 1 July to 15 August 2022, based on the China Life Care Association. In the first stage, four regions—northeast, east, central, and west—were selected. Subsequently, specific provinces or municipalities within these regions were identified. A total of 30 provinces were chosen based on the regional distribution of hospital members affiliated with the China Life Care Association. The sample size for each province was determined proportionally to its population relative to the national population. In the second stage, the number of tertiary hospitals to be included from each province was calculated, taking into account the population distribution across cities within the province and the annual number of inpatients per hospital. This ensured a representative number of patients per hospital for the survey. In the third stage, hospital managers from the selected hospitals in the second stage served as the sampling units. Ultimately, a total of 83 hospitals were included in the study. Figure 1 presents a schematic diagram illustrating the process employed in this study.

### 2.2 Instruments and measures

The survey instruments were largely based on the Methodist Health Care System Nurse Caring Instrument (NCI) developed by the Nursing Care Quality Control Council of the Houston Health Care System in 2000 to assess inpatients' satisfaction with humanistic nursing (17). The survey comprised two sub-questionnaires, which were developed and approved by the research team based on a comprehensive literature review (18). The general information questionnaire included the patient's hospital, sex, age, marital status, number of children, education attainment, place of residence, monthly household income, whether it was the patient's first visit, time of visit, type of medical insurance, department visited, area of visit, and whether surgery was performed. The Humanistic Nursing Care Satisfaction Scale was assessed using the NCI, which was developed by the Nursing Care Quality Control Council of the Houston Health Care System as part of the Humanistic Care Satisfaction questionnaire (19–21). This questionnaire is the most frequently used instrument for assessing the quality of care and consists of 20 items covering 12 dimensions: care coordination, competence, teaching/learning, emotional support, respect for individuality, physical comfort, availability, helping/trusting relationships, patient/family involvement, physical environment, spiritual environment, and outcomes. The items reflect concepts from the caring literature and the caring theory of Watson and others, as well as items that are familiar areas of assessment on other instruments. Each item provides seven answers corresponding to a seven-point Likert scale. The items (“seldom or rarely,” “often or frequently,” “always or almost always,” and “does not apply”) were scored from one to seven, resulting in a total score of 140. A higher score indicates greater satisfaction with nurses' care.

After obtaining authorization from the original authors, we embarked on a comprehensive adaptation and reliability testing process. Our team meticulously employed a multifaceted scientific approach to develop, localize, and validate the questionnaire's reliability, feasibility, and acceptability. This robust process encompassed an extensive literature review, patient cognitive interviews, input from a diverse range of stakeholders (including healthcare regulators, hospital managers, doctors, nurses, and patients), psychometric analyses, pilot tests conducted across three provinces, small-scale multidisciplinary expert consultations, and field tests. To ensure the feasibility and acceptability of the tool, we analyzed missing item response percentages, reviewed interviewer-reported acceptability, and assessed the time and ease of administration. The internal consistency and reliability of each dimension were evaluated using Cronbach's α coefficients and inter-subscale correlations. The Chinese version of the NCI questionnaire demonstrated excellent reliability with an overall Cronbach's alpha of 0.982. The helping/trusting dimension obtained a Cronbach's α of 0.943, the respect for individuality dimension a Cronbach's α of 0.907, the patient/family involvement dimension a Cronbach's α of 0.937, the emotional support dimension a Cronbach's α of 0.895, and the care coordination dimension a Cronbach's α of 0.908. For the remaining dimensions, which consisted of single items, Cronbach's α coefficients could not be calculated. Overall, the questionnaire's strong reliability was well-supported within the context of our study, underscoring a coherent, logical, and methodically sound research design.

### 2.3 Sample size calculation

The survey's target population included inpatients admitted to large public tertiary hospitals. The inclusion criteria for participation were: (1) patients who were stably residing in rural areas of China, whether they paid rural health insurance or not; (2) patients who were older than 18 years; (3) patients who were hospitalized for >5 days; (4) patients who were conscious; and (5) patients who had voluntarily completed the questionnaire. The exclusion criteria were: (1) patients who were unable to use a smartphone or have intellectual or cognitive impairments to complete the cognitive assessments required for the trial; and (2) patients without smartphones or who were unable to answer the questionnaire using a smartphone.

Drawing on patient satisfaction rates reported for outpatient and inpatient settings in both China and internationally, we established the expected satisfaction rate at 70% (22–25). The primary formula used for determining the study's sample size was *n* = ${\text{u}}_{\alpha /2}^{2}$ (1-π)/δ^2^, where u_α/2_ = 1.96, α = 0.05, π represents anticipated patient satisfaction with humanistic nursing (70% in this study), and δ denotes the admissible error (10% for this study). Using this formula, the theoretical sample size was calculated as 90, with an additional 10% added to account for any potential loss during the study (26). After taking into account variations in the number of hospital inpatients and to make the data more representative, we decided to enroll a minimum of 200 patients from each tertiary hospital. The total number of patients interviewed for this study was 13,325. To control for selection bias, a final sample of 8,263 participants was included in the analysis after excluding incomplete or invalid questionnaires.

### 2.4 Data collection

The Human Care Professional Committee of the China Life Care Association initiated and coordinated the study to ensure high-quality data collection and analysis. The research was carried out utilizing the Questionnaire Star Platform. Following the acquisition of consent from hospital administrators, leaders from diverse hospitals nationwide, identified as research subjects, were briefed on the questionnaire's purpose and specific implementation details before the commencement of the nationwide survey. Trained researchers conducted a random-sampling survey of eligible rural inpatients at the hospital's discharge settlement office, after obtaining informed consent for their participation in the study and administering the questionnaire survey. All the survey questionnaires were completed and submitted anonymously. Rigorous quality control measures were instituted to uphold the scientific and authentic nature of data collection. The survey's data collectors received comprehensive training from various hospitals across the country before the nationwide survey. The training encompassed research objectives, significance, target population, and questionnaire completion methods to ensure the scientific and authentic nature of data collection. Throughout the questionnaire survey, strict adherence to the inclusion and exclusion criteria was maintained in the selection of research subjects. Patients were briefed on the purpose of the research, with an assurance of strict adherence to confidentiality and anonymity principles. Quality control measures involved scrutinizing the questionnaires' quality and excluding invalid questionnaires before data entry. Simultaneous input by two individuals during data entry was employed to ensure accuracy and precision.

### 2.5 Statistical analysis

The statistical analysis was conducted after excluding invalid questionnaires obtained from Questionnaire Star. The data were imported into SPSS (version 25.0) using an Excel spreadsheet. To ensure accuracy, a two-person crosscheck method was employed after eliminating invalid questionnaires. Continuous variable distributions were described using means and standard deviations, whereas categorical variable distributions were described using frequency counts. Univariate methods were initially used to analyze the relevant indicators in order to identify risk factors, which were subsequently incorporated into a multivariate logistic regression analysis.

## 3 Results

### 3.1 Participants' characteristics

A total of 13,225 patients were interviewed for this study, of which 8,263 patients had valid questionnaires; 4,962 patients' questionnaires were declared invalid and excluded, mostly due to incomplete responses. The 8,263 patients included in the study were distributed across 50 tertiary hospitals in 25 provincial administrative units across the country. The sample consisted of 4,328 (52.38%) male patients and 3,935 (47.62%) female patients, of which 5,679 (68.73%) were between 50 and 70 years old, 5,285 (63.96%) were married, and 7,510 (90.88%) had one or more children. Patients' literacy levels were generally concentrated at a high school level or below (7,049; 85.31%). Most were mainly engaged in agricultural production, employment, or contract work (5,329; 64.49%), the majority mainly resided in rural areas (5,568; 67.38%), and most had a monthly household income of < 5,000 yuan (6,171; 74.68%). The purpose of their visit was mainly for internal medical or surgical treatment (6,916; 83.70%); 6,962 (83.82%) of the patients had rural co-operative medical insurance and 6,883 (83.30%) of them were visiting a large hospital for the first time. The patients' demographic characteristics are summarized in Table 1.

**Table 1:** Comparison of humanistic caring satisfaction scores of subjects with different demographic characteristics.

**Project**	**Grouping**	**Numbers**	**Score (*x* ±*s*)**	**Statistics**	***P*-value**
Overall	–	–	81.62± 16.85	–	–
Gender	Male	4,328	82.23 ± 13.29	16.28^a^	0.328
	Female	3,935	81.32 ± 15.68		
Age	18–30	453	93.23 ± 23.68	71.23^b^	0.022
	31–40	631	86.75± 13.85		
	41–50	526	85.91± 16.33		
	51–60	2,365	80.84± 16.27		
	61–70	3,214	80.32± 22.52		
	71–80	527	78.66 ± 19.36		
	≥81	547	76.32± 16.85		
Marital status	Married	5,285	86.25 ± 16.28	32.55^b^	0.039
	Unmarried	795	83.22 ± 17.87		
	Divorced/separated	525	79.62 ± 21.39		
	Widowed	1,658	76.21± 16.82		
Children	No child	653	77.21 ± 17.83	36.57^b^	0.036
	1 child	3,225	80.23 ± 10.29		
	≥2 children	4,385	84.03 ± 13.12		
Educational attainment	Primary school	1,536	75.58 ± 10.89	53.24^b^	0.026
	Junior high school	2,912	80.23 ± 12.39		
	High school/technical Secondary school	2,601	85.39 ± 12.28		
	College	862	90.82 ± 13.68		
	Bachelor degree or above	352	86.25 ± 15.62		
Occupation	Farmer	2,443	73.25 ± 12.35	57.98^b^	0.023
	Worker	825	86.95 ± 15.81		
	Military person	336	90.24 ± 22.69		
	Leader	0	–		
	Employed	1,361	81.07 ± 17.38		
	Self-employed	952	82.73 ± 18.08		
	Freelance	1,525	82.37 ± 15.99		
	Retired	0	–		
	Student	538	90.85 ± 17.97		
	Other	283	78.48 ± 15.36		
Place of residence	City	712	83.74 ± 18.92	19.68^b^	0.229
	Town	1,983	81.92 ± 15.36		
	Rural	5,568	77.28 ± 13.28		
Monthly household income (yuan)	< 3,000	2,386	79.38 ± 13.33	68.85^b^	0.018
	3,000– < 5,000	3,785	78.32 ± 16.86		
	5,000– < 8,000	1,257	86.32 ± 16.87		
	8,000– < 10,000	1,035	91.07 ± 19.69		
	>10,000	428	93.23 ± 15.54		
Department visited	Internal medicine department	3,089	81.32 ± 17.00	50.31^b^	0.031
	Surgical	3,827	81.12 ± 16.60		
	Obstetrics and gynecology	763	80.58 ± 18.28		
	Pediatric	0	–		
	Intensive care medicine	51	73.41 ± 15.82		
	Other	533	80.37 ± 16.23		
Medical insurance type	Rural cooperative medical insurance	6,962	79.35 ± 16.99	45.96^b^	0.035
	Basic medical insurance scheme for urban employees	0	–		
	Basic medical insurance scheme for urban residents in urban areas	0	–		
	Commercial insurance	632	86.39 ± 18.32		
	Public expense	0	–		
	Own expense	359	75.35 ± 13.86		
	Other	310	80.87 ± 18.63		
Region	Northeast China	928	83.21 ± 16.49	10.17^b^	0.443
	North China	1,353	83.86 ± 18.32		
	East China	1,607	80.83 ± 15.62		
	Middle China	1,354	81.22 ± 19.32		
	West China	986	80.28 ± 15.36		
	Northwest China	823	79.29 ± 18.95		
	South China	1,212	79.33 ± 18.32		
First-time visitor	Yes	6,883	79.85 ± 18.36	66.35^a^	0.019
	No	1,380	85.33 ± 17.59		
Surgical patient	Yes	5,035	81.52 ± 18.36	7.22^a^	0.573
	No	3,228	83.05 ± 17.85		

^a^t-value.

^b^F-value.

### 3.2 Humanistic nursing care satisfaction scores of patients with diverse characteristics

Table 1 summarizes the results of the comparative analysis of humanistic care satisfaction scores based on patients' characteristics. The Humanistic Care Satisfaction Scale's maximum score is 140, and the overall mean satisfaction score for humanistic care in this study was 81.62 ± 16.85, showing that satisfaction with nursing humanistic care among rural Chinese patients attending large tertiary public hospitals was generally at a low level. We found no statistical difference in satisfaction with humanistic care among patients by gender, place of residence, region, and whether or not they had surgery, while age, marital status, number of children, educational attainment, occupation, monthly household income, department visit, medical insurance type, and first-time visitor showed statistical differences (Table 1).

Specifically, the older the patient, the lower their satisfaction with humanistic care, and the lowest level of satisfaction with humanistic care was among patients over 80 years old (76.32 ± 16.85). Marital status also had a greater impact on patients' satisfaction, with the lowest level of satisfaction with humanistic care among widowed patients (76.21 ± 16.82 points). We also found a direct correlation between number of children and the patient's satisfaction with humanistic care. In terms of education attainment, rural patients with a primary school education had the lowest level of satisfaction with humanistic care (75.58 ± 10.89). In terms of occupation, farmer had the lowest level of satisfaction with humanistic care (73.25 ± 12.35). In terms of monthly household income, those with an income of < 3,000 and 3,000–5,000 had the lowest satisfaction with humanistic care. First-time visitors also had lower levels of satisfaction with humanistic care (Table 1).

### 3.3 Factors associated with humanistic care satisfaction

The multiple linear regression analysis (Table 2) revealed significant associations between various factors and humanistic care satisfaction scores. The significant variables included in the model were selected based on their significance in the univariate analysis to ensure clarity and methodological transparency. Children number (β = 0.363, SE = 0.076), education attainment (β = 0.285, SE = 0.062), monthly household income (β = 0.391, SE = 0.061), and first time visited (β = 0.338, SE = 0.039) were found to have significant positive correlations with humanistic care satisfaction scores. In contrast, age (β = −0.232, SE = 0.051), marital status (β = −0.135, SE = 0.039) and department visited (β = 0.383, SE = 0.114) displayed significant negative correlations with humanistic care satisfaction scores. The *R*^2^ value for this analysis was 0.592 and the adjusted *R*^2^ value was 0.376.

**Table 2:** Multiple linear regression analysis of the factors associated with humanistic care satisfaction scores.

**Variable**	**β**	**SE**	***P***	***R* ^2^**	**Adjusted *R*^2^**
Age	−0.232	0.051	0.036	0.592	0.376
Marital status	−0.135	0.029	0.039		
Number of children	0.363	0.076	0.028		
Educational attainment	0.285	0.062	0.033		
Occupation	0.265	0.032	0.568		
Monthly household income	0.391	0.061	0.027		
Department visited	0.383	0.114	0.353		
Medical insurance type	0.274	0.029	0.237		
First-time visitor	0.338	0.039	0.043		

## 4 Discussion

This national cross-sectional study's findings shed light on rural patients' satisfaction levels with humanistic nursing care at large public tertiary hospitals in China. The emphasis on humanistic care in healthcare settings has gained significance globally, and its impact on patient satisfaction is evident in various contexts. In rural healthcare settings in China, where challenges related to access, communication, and cultural differences are pronounced, understanding the nuances of humanistic nursing care is critical for improving healthcare outcomes (27–30). Our findings revealed that the overall satisfaction with humanistic nursing care among rural patients was moderately low, emphasizing a critical area for improvement within the healthcare system.

One significant finding was the influence of age on satisfaction levels. Older patients, particularly those over 80, reported the lowest satisfaction scores. This could be attributed to the increased vulnerability and higher expectations of this age group for empathetic and compassionate care. Additionally, older adult patients may face more chronic conditions and complex health issues, increasing their need for comprehensive care, which the current system might not fully meet. Older adult patients, particularly those of advanced age, often encounter significant challenges due to the prevalence of multiple chronic conditions, limited income, and lower educational attainment. These factors can lead to confusion and difficulty in navigating the healthcare system, especially in large tertiary hospitals. To address these issues, the government has implemented targeted measures for older adult patients in rural areas, including family doctor services, major illness insurance, medical assistance programs, and a long-term care insurance system. At the hospital level, efforts such as training ward and outpatient nurses in humanistic care and establishing humanistic care settings in patient wards have been undertaken (31–34). These initiatives aim to continuously improve patient satisfaction with humanistic care and ensure a more supportive healthcare experience. Marital status emerged as another crucial factor, with widowed patients reporting lower satisfaction levels. This demographic may experience heightened feelings of loneliness and lack of support, thus increasing their need for humanistic care. The loss of a spouse can result in a significant emotional support gap, leading to greater dependence on the healthcare system (35). Hospitals should consider implementing support systems and counseling services tailored to widowed patients to improve their overall healthcare experience.

Educational attainment and occupation were also significant determinants of satisfaction. Patients with lower educational levels and those engaged in farming reported lower satisfaction scores. These findings suggest that educational background and socioeconomic status may influence patients' perceptions and expectations of care. Patients with lower educational levels might lack understanding of medical information, affecting their perception of care quality. Farmers and low-income workers might feel overlooked and unfairly treated due to economic pressures and lack of social support. Efforts to enhance communication efforts to enhance communication and understanding between healthcare providers and patients with diverse educational and occupational backgrounds could lead to improved satisfaction. Over the years, national policies to support rural patients and low-income groups have become increasingly robust. Medical insurance reimbursement for rural patients has steadily expanded, and local governments often provide financial subsidies for low-income individuals, which reduces insurance premiums and alleviates the financial burden on rural patients (36–38). At the hospital level, several strategies have been implemented to improve satisfaction among rural patients. Humanistic care training is provided to healthcare staff to enhance communication skills and understanding of patients' psychological and emotional needs, fostering a more empathetic and personalized approach to patient care (39, 40). Guidance and navigation services are available to assist first-time visitors or patients unfamiliar with hospital procedures, helping rural patients locate relevant departments and understand the treatment process, thereby reducing anxiety and confusion. In collaboration with community health initiatives, some hospitals provide family doctor services for rural patients, ensuring continuity in health management and support before and after hospital visits. Additionally, specialized reception desks and consultation centers have been established to assist rural patients, answer questions, and offer personalized medical guidance. To address language and cultural barriers, patient services may incorporate local dialects or multiple languages, enabling rural patients from diverse backgrounds to understand medical information more effectively. Hospitals have also implemented humanistic care enhancements in hospital wards, creating a comfortable environment and providing psychological support and social work services to ensure that patients and their families feel supported during their stay. Financial counseling and assistance programs are available for economically disadvantaged rural patients, helping them navigate reimbursement procedures and access fee reduction policies to alleviate financial stress. Furthermore, health education initiatives, including lectures, brochures, and information dissemination, aim to inform rural patients about disease prevention and management, thereby improving their self-management capabilities. Collectively, these measures are designed to enhance the overall experience and satisfaction of rural patients during their medical treatment and care, contributing to a more inclusive and supportive healthcare environment.

Interestingly, first-time visitors to tertiary hospitals reported lower satisfaction levels compared to those with prior visits. This could be due to unfamiliarity with the hospital environment and processes, leading to increased anxiety and discomfort. First-time visitors may not be familiar with hospital procedures and facilities, which can add to their stress and anxiety. Hospitals should consider implementing orientation programs and support services for first-time visitors to help them navigate the healthcare system more comfortably. Income levels also played a significant role in determining satisfaction. Patients with lower monthly household incomes reported lower satisfaction scores, highlighting the impact of economic disparities on healthcare experiences. Low-income patients may face greater financial burdens and resource limitations, leading to lower expectations and satisfaction with healthcare services. Ensuring equitable access to high-quality humanistic care regardless of economic status is essential for improving overall patient satisfaction. The department visited by patients was another important factor influencing satisfaction. Departments that primarily deal with acute or severe medical conditions, such as intensive care units, reported lower satisfaction scores. This may be due to the high-stress environment and the critical nature of care provided in these settings. Patients in intensive care units often have severe conditions and higher demands for care quality, while healthcare providers in these environments might prioritize technical operations over emotional support. Enhancing humanistic care practices in such departments, including providing emotional support and clear communication, could improve patient satisfaction. Our study also found that the type of medical insurance held by patients influenced their satisfaction levels. Patients with rural cooperative medical insurance reported lower satisfaction compared to those with commercial insurance. This disparity may reflect differences in the perceived quality of care and financial burdens associated with different insurance types. The coverage and reimbursement rates of rural cooperative medical insurance may be lower than those of commercial insurance, resulting in greater financial pressure for patients. Addressing these disparities through policy changes and improving the coverage and benefits of rural cooperative medical insurance could enhance patient satisfaction.

### 4.1 Limitations

This study has several limitations that should be acknowledged. First, we did not conduct an in-depth analysis of each of the 12 dimensions of the NCI questionnaire, such as care coordination and emotional support. Due to the potential overlap between these dimensions, a detailed examination could introduce complexity and confusion. To maintain clarity and focus, we opted for an overall assessment of humanistic care. However, this approach may limit a deeper understanding of specific aspects of care. Future research could address this limitation by exploring each dimension individually, offering more granular insights into the components of humanistic nursing care.

Second, the cross-sectional design of this study precludes the establishment of causal relationships between patient characteristics and satisfaction with humanistic care. Longitudinal studies would be valuable in tracking changes over time, providing a more dynamic understanding of how various factors influence patient satisfaction. Third, there is a potential for selection bias, as participants who completed the survey and provided qualified data may have relatively higher educational and economic levels. This could result in an overestimation of rural residents' satisfaction with their medical visits. Future studies should aim to minimize this bias by employing more inclusive sampling strategies. Finally, this study focused exclusively on rural patients in large public tertiary hospitals in China. As a result, the findings may not be generalizable to other healthcare settings or patient populations. Future research should consider diverse healthcare environments and include a broader range of patient demographics to enhance the generalizability and applicability of the results.

## 5 Conclusions

This study presents a comprehensive evaluation of humanistic nursing care satisfaction among rural patients and elucidates the various factors that contribute to their perceptions of healthcare services. The outcomes of this cross-sectional investigation are poised to serve as a cornerstone for evidence-based recommendations, strategically designed to elevate the caliber of healthcare services tailored to the unique needs of rural patients in China. Older adults, widowed individuals, and first-time patients expressed lower levels of satisfaction, highlighting the need for tailored interventions. Factors such as education and family support play an important role in improving patient satisfaction with humanistic nursing. This national cross-sectional study significantly advances our understanding of the determinants influencing humanistic care satisfaction among rural patients within the context of China's public tertiary hospitals. The identified associations not only offer valuable insights but also lay the groundwork for targeted interventions and policy formulations and, ultimately, the overall quality of healthcare delivery.

## Data Availability

The raw data supporting the conclusions of this article will be made available by the authors, without undue reservation.
